# Large-scale analysis of antigenic diversity of T-cell epitopes in dengue virus

**DOI:** 10.1186/1471-2105-7-S5-S4

**Published:** 2006-12-18

**Authors:** Asif M Khan, AT Heiny, Kenneth X Lee, KN Srinivasan, Tin Wee Tan, J Thomas August, Vladimir Brusic

**Affiliations:** 1 The Division of Biomedical Sciences, Johns Hopkins Singapore, 31 Biopolis Way, #02-01 The Nanos, Singapore 138669, Singapore; 2 Department of Microbiology, The Yong Loo Lin School of Medicine, National University of Singapore, 5 Science Drive 2, Singapore 117597, Singapore; 3 Department of Biochemistry, The Yong Loo Lin School of Medicine, National University of Singapore, 5 Science Drive 2, Singapore 117597, Singapore; 4 Department of Pharmacology and Molecular Sciences, The Johns Hopkins University School of Medicine, 725 North Wolfe Street, Baltimore, MD 21205, USA; 5 School of Land and Food Sciences, and Institute for Molecular Biosciences, University of Queensland, Brisbane, QLD 4072, Australia

## Abstract

**Background:**

Antigenic diversity in dengue virus strains has been studied, but large-scale and detailed systematic analyses have not been reported. In this study, we report a bioinformatics method for analyzing viral antigenic diversity in the context of T-cell mediated immune responses. We applied this method to study the relationship between short-peptide antigenic diversity and protein sequence diversity of dengue virus. We also studied the effects of sequence determinants on viral antigenic diversity. Short peptides, principally 9-mers were studied because they represent the predominant length of binding cores of T-cell epitopes, which are important for formulation of vaccines.

**Results:**

Our analysis showed that the number of unique protein sequences required to represent complete antigenic diversity of short peptides in dengue virus is significantly smaller than that required to represent complete protein sequence diversity. Short-peptide antigenic diversity shows an asymptotic relationship to the number of unique protein sequences, indicating that for large sequence sets (~200) the addition of new protein sequences has marginal effect to increasing antigenic diversity. A near-linear relationship was observed between the extent of antigenic diversity and the length of protein sequences, suggesting that, for the practical purpose of vaccine development, antigenic diversity of short peptides from dengue virus can be represented by short regions of sequences (~<100 aa) within viral antigens that are specific targets of immune responses (such as T-cell epitopes specific to particular human leukocyte antigen alleles).

**Conclusion:**

This study provides evidence that there are limited numbers of antigenic combinations in protein sequence variants of a viral species and that short regions of the viral protein are sufficient to capture antigenic diversity of T-cell epitopes. The approach described herein has direct application to the analysis of other viruses, in particular those that show high diversity and/or rapid evolution, such as influenza A virus and human immunodeficiency virus (HIV).

## Background

Dengue virus has four serotypes (DV1, DV2, DV3 and DV4) that show substantial genetic diversity both within and between serotypes. Sequence comparison studies showed 30–40% difference in amino acid sequences between serotypes [1,2]. The amino acid differences within each serotype are lower but the observed intra-serotype diversity is sufficiently large to warrant the definition of clusters of dengue virus variants [3,4]. Studies of genetic diversity have focused on clade diversity and replacement [4], mutation spectra [5], conserved regions [6] and implications for clinical manifestations [3]. Several studies have focused on the analysis of antigenic diversity (diversity of targets of immune responses in protein sequences) of dengue virus; these studies focussed on experimental mapping of antibody recognition sites [7-11] and T-cell epitopes [2,12-15] and subsequent analysis of their diversity. Recently, Simmons *et al*. [15] analyzed the T cell responses of individuals infected with DV2 by ELISpot assay and identified 34 peptides of several dengue proteins as potential novel T-cell epitopes.

Generally, there is a correspondence between genetic and antigenic evolution of viruses, but genetic changes may result in disproportionately large antigenic changes [16,17]. While genetic and antigenic diversity in dengue virus strains had become evident [18], large-scale and detailed systematic analyses that explore their relationship have not been reported. Understanding this relationship is important for the study of vaccine development, especially in rapidly mutating viruses. In this paper, we will focus on protein sequence diversity, and thus consider only genetic variations that affect the protein sequences.

Biological studies of antigenic diversity require great experimental effort, even for a single viral protein. Consequently, most research groups focus on studying small number of viral sequences. Rapid accumulation of sequence data from both classical and genomic/proteomic approaches makes the experimental studying of antigenic diversity difficult and time-consuming. A bioinformatics approach is necessary to support large-scale antigenic analysis of viral diversity, which can complement laboratory experiments.

In this study, we have developed a bioinformatics method to analyze antigenic diversity in the context of T-cell mediated immune responses. We studied antigenic diversity of more than 9000 dengue virus protein sequences reported in the NCBI Entrez protein database [19]. The study aimed to identify a minimal set of sequences that encodes the complete antigenic diversity of short peptides from all known sequences in dengue virus serotypes. Short peptides, principally 9-mers were studied because they represent the predominant length of binding cores of T-cell epitopes. We analysed the relationship between short-peptide antigenic diversity and protein sequence diversity of dengue virus; the analysis was performed at two time points to help understand the effects of the accumulation of sequence data to the relationship. We have also analyzed the effects of sequence determinants on antigenic diversity of short peptides. This study provides a framework for large-scale, systematic analysis of antigenic diversity for the protein sequences of any virus. We did not analyze B-cell epitope antigenic diversity because of their complex conformational nature. Although linear B-cell epitopes exist and our method can be used to study them, very often, they also show conformational preferences and dependence on the context of a protein antigen [20]. Further, only approximately 10% of B-cell epitopes from native proteins are linear [21].

## Results

### Dengue serotype protein datasets

Data of June 2004 (Table 1), collected from the NCBI Entrez protein database, contained a total of 3699 sequences representing the ten proteins encoded by the genomes of the four serotypes (Table 2). The number of these reported sequences increased nearly three-fold during the following 18 months (9512 sequences; see Table 1). The removal of duplicates (identical protein sequences) reduced these collected sequences to 1318 (2004) and 2419 (2005) unique sequences (Table 1). More than 64% of the sequences collected in 2004 were identical and redundant, and this redundancy increased by approximately 10% in 2005 (75%). The number of reported unique sequences varied greatly among the proteins, ranging from 69 NS4a to 998 E sequences in 2005 set (Table 3). Minor errors of annotation, mainly of the cleavage sites, were corrected prior to analysis for 17 sequences (see additional file 1: Table S1.pdf).

### Intra- and inter-serotype amino acid sequence identities of dengue proteins

Earlier studies of dengue proteins, mainly E and NS1 [1,22-25], have shown substantial amino acid sequence diversity both within and between the serotypes. In our study, we surveyed the extent of amino acid variation and conservation among dengue viruses by calculating pairwise percentage amino acid identity of unique sequences for each dengue protein, intra- and inter-serotype, using the large dengue data set of 2005. The intra- and inter-serotype percentage sequence identities (PSI) for all dengue proteins are shown in Table 4.

The intra-serotype percentage sequence identity was between 92% and 99%, except for C, pM, E and NS1 of DV2, which showed minimum sequence identities ranging from 79% to 89%. In contrast, the average inter-serotype percentage sequence identity was in the range of 60–79%, except for NS2a. The NS3, NS4b and NS5 proteins are highly conserved across the serotypes, with average sequence identities in the range of 77–79%, probably because of their involvement in forming the RNA replication complex [26]. The NS2a protein is the most diverse across the serotypes (average PSI of 39%), although it is highly conserved within each serotype. The inter-serotype diversity observed for NS2a is comparable to the inter-*Flavivirus *diversity of the envelope protein, which shows approximately 40% amino acid identity [27].

### Minimal sequence sets representing dengue virus antigenic diversity

**Figure 1:**
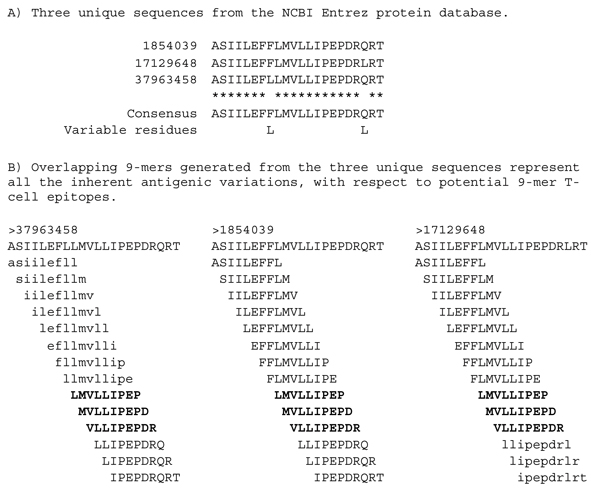
**Definition of antigenically redundant sequence**. A) The three sequences (NCBI GI no.: 1854039, 17129648 and 37963458) are each unique, and residues that vary among them are shown. B) Overlapping 9-mers generated from the three unique sequences represent all the inherent antigenic variations, with respect to potential 9-mer T-cell epitopes. Although the three sequences are each unique, they share identical 9-mers. 9-mers shown in uppercase are those with an identical match in two of the unique sequences analyzed, while those in bold uppercase have an identical match in all three sequences; unique 9-mers are shown in lowercase. All the 9-mers in sequence 1854039 have a match in at least one of the other two sequences; thus, the antigenic diversity of this sequence can be covered by the other two sequences combined, rendering the sequence 1854039 antigenically redundant. Hence, the minimal number of sequences required to represent antigenic diversity for this dataset is two.

In addition to identical protein sequences, another source of sequence redundancy, relative to this study, is the presence of antigenically redundant sequences. These sequences exist because of the identity of many amino acid residues among the individually unique protein sequences (see Results section sub-heading: *Intra- and inter-serotype amino acid sequence identities of dengue proteins*), resulting in the presence of targets of T-cell mediated immune responses (T-cell epitopes) that are identical among viral variants. Antigenically redundant sequences can be removed without loss of information on antigenic diversity among the sequences sets. For example, if in a dataset of three sequences, all of the overlapping 9-mers in one sequence have a match in at least one of the other two sequences, the antigenic diversity of this sequence can be covered by the other two sequences combined, thus rendering the first sequence antigenically redundant (Figure 1).

The removal of antigenically redundant sequences using our bioinformatics method (see Methods section sub-heading: *Protein sequence and antigenic diversity analysis of dengue virus*) resulted in a further reduction of the number of dengue unique sequences to a total of 969 (2004 set) or 1684 (2005 set). Those two sets represent the complete antigenic diversity of short peptides for all four dengue serotypes (Table 5). The increase in the number of unique sequences required to represent the complete antigenic diversity of short peptides in the four dengue serotypes in 2005 (compared to 2004) is an indication that more short-peptide antigenic diversity was found in the new sequences accumulated in the database. However, the percentage of unique sequences required to represent the complete short-peptide antigenic diversity of all four dengue serotypes in 2005 decreased (from 74% in 2004 to 70% in 2005) because of an increase in antigenic redundancy. This observation indicates that the increase in the number of unique protein sequences (representing protein sequence diversity) deposited in public databases is generally accompanied by a slower increase in short-peptide antigenic diversity.

### Characterization and application of sequence variables that affect antigenic diversity

We examined the effects of sequence determinants, such as number and length of sequences, on the short-peptide antigenic diversity of dengue virus. These analyses were carried out using test datasets of different numbers of sequences (20, 40, 60, 80, 100, 120 and 140 sequences) and different lengths (23, 46, 128, 138, 276 and 460 aa) that were randomly selected from a set of DV2 envelope protein sequences with repeated sampling for 20 times. Antigenic diversity analysis of each test dataset was performed to identify a minimal set of sequences that represents the complete short-peptide antigenic diversity for each dataset. These minimal sets were used to analyze the effects of the sequence determinants on antigenic diversity.

#### Effects of number of sequences on short-peptide antigenic diversity

**Figure 2:**
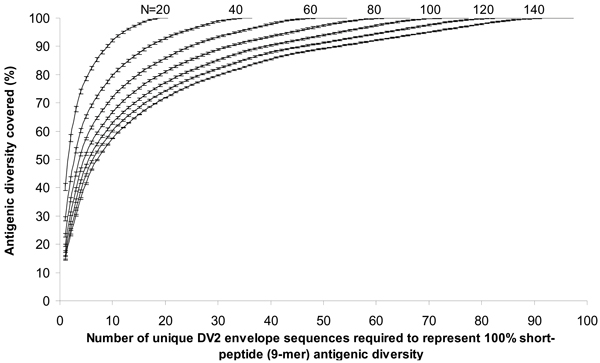
**Short-peptide (9-mer) antigenic diversity as a function of number of sequences**. Short-peptide antigenic diversity has an asymptotic relationship to number of unique dengue virus serotype 2 (DV2) envelope sequences (N). Each curve shows the cumulative percentage coverage of short-peptide antigenic diversity. Vertical bars represent standard error for repeated random sampling of 20 times.

An increase in the number of unique sequences in a dataset reduces the fraction required to represent the complete short-peptide antigenic diversity (Table 6). This observation reflects an asymptotic relationship between the number of unique sequences and the percentage of the complete short-peptide antigenic diversity that is covered (Figure 2). Asymptotic curves were observed for all proteins of the four dengue serotypes (data not shown). The shape of the curve indicates that a single sequence will cover only a small proportion of the total short-peptide antigenic diversity and that for proteins with a large number of unique sequences, the addition of a single new variant sequence has little effect on the overall antigenic diversity.

#### Effects of length of sequences on short-peptide antigenic diversity

**Figure 3:**
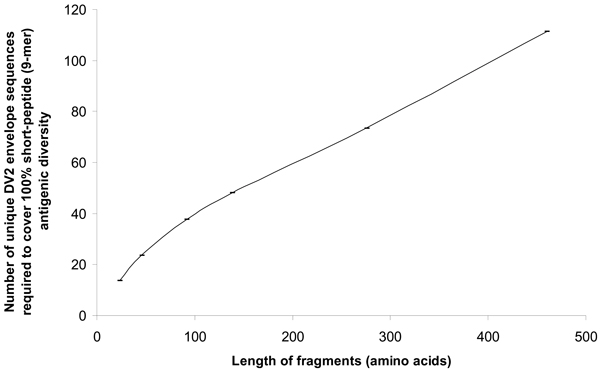
**Short-peptide (9-mer) antigenic diversity as a function of length of sequences**. Short-peptide antigenic diversity shows a linear relationship to the sequence length of dengue virus serotype 2 (DV2) envelope protein. Vertical bars represent standard error for repeated random sampling of 20 times.

A decrease in the length of sequences of a dataset reduces the fraction required to represent the complete short-peptide antigenic diversity of the dataset (Table 7). This reduction was achieved by removal of two types of redundancy: identical fragments and antigenically redundant fragments. The number of identical fragments increases significantly with a decrease in the length of the fragments because of the limited variability associated with smaller size. Hence, the effect of sequence length is significant, especially for very short fragments (23 aa), for which only ~7% of the unique fragments were required to represent complete antigenic diversity of the short fragments (a reduction of ~93%). Overall, the results indicate that short-peptide antigenic diversity has a near-linear relationship to sequence length (Figure 3).

## Discussion

In this study, we applied a systematic bioinformatics approach to collect, clean, organize and analyze the antigenic diversity of short peptides in reported protein sequence data of dengue virus. We have developed a computational method for the analysis of antigenic diversity in the context of T-cell mediated immune responses. The method was applied for the analysis of short-peptide antigenic diversity of dengue virus to determine a minimal sequence set that encodes the complete antigenic diversity of linear epitopes within each dengue virus serotype. We studied the relationship between short-peptide antigenic diversity and protein sequence diversity of DV and also explored the effects of sequence determinants on viral antigenic diversity. Our analysis showed that the minimal number of unique sequences required to represent complete antigenic diversity of linear epitopes in dengue virus is significantly smaller than that required to represent complete protein sequence diversity. Short-peptide antigenic diversity shows an asymptotic relationship to the number of unique sequences and linear relationship to the length of protein antigens.

**Figure 4:**
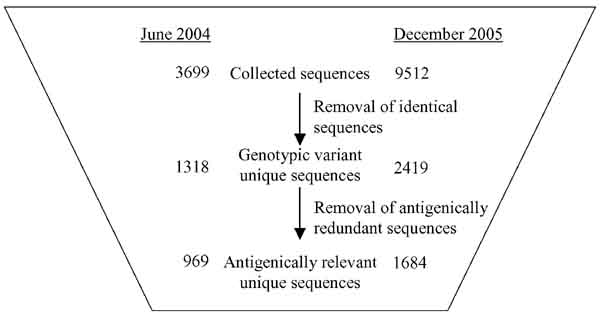
Flowchart summarizing the steps undertaken to identify the antigenically relevant unique sequence for dengue virus.

The minimal sequence set that encodes the complete short-peptide antigenic diversity for each dengue virus serotype was derived through removal of identical sequences and antigenically redundant sequences (Table 5 and Figure 4). Both reductions occurred without any loss of information on antigenic diversity among the sequences. The largest reduction was accomplished through the removal of identical sequences, since only 36% (year 2004) or 25% (year 2005) of the sequences were unique. The identical sequences originated from dengue virus strains that were unique variants with respect to the whole polyprotein, but were identical to other dengue strains with respect to individual proteins, resulting in many duplicate protein sequences. The removal of antigenically redundant sequences also involved a significant proportion of the sequences, approximately one-third of all unique sequences (2004: 26%; 2005: 30%), reflecting the high antigenic redundancy among the dengue virus variants, which often differed by only a few amino acids. Despite significant reduction achieved by reducing the collected sequences to minimal sequences, a large number of protein sequences, 969 in 2004 and 1684 in 2005, were still required to represent the complete short-peptide antigenic diversity of dengue virus.

It is clear that antigenic diversity in the reported dengue sequences is large. With many asymptomatic human and animal carriers of dengue viruses representing a huge reservoir for emergence of new strains [6,24,28], the diversity is expected to increase, although at a progressively slower pace. This is because antigenic redundancy increases when the number of sequences increases; we observed that when the dataset for a particular protein reaches approximately 200 sequences, the effect of addition of new sequences to increasing antigenic diversity is marginal.

Our study of factors that affect antigenic diversity provided insight into dealing with the increasing T-cell epitope antigenic diversity in the context of vaccine development. Length of sequences had the largest effect on short-peptide antigenic diversity. The asymptotic behaviour of antigenic diversity increase was observed for the increase in the number of sequence variants. For practical purposes of vaccine formulation, antigenic diversity cannot be represented by whole protein sequences because it is not feasible to use these sequences for systematic experimental analysis: they are long and their number is increasing rapidly. The implication is that conventional vaccination strategies, which utilize whole attenuated pathogen with little knowledge of the specificity of immune responses they elicit, may not be suitable for providing protection from multiple variants of viruses. Furthermore, it may be difficult to optimize such vaccine according to the human leukocyte antigen (HLA) profile of the population receiving the vaccine [29,30], as neither the identities of the HLA molecules that bind T-cell epitopes, nor the epitopes themselves are known.

The more effective vaccine strategy that we propose is to focus on short segments of proteins (~<100 aa) that are known to be specific targets of immune responses (such as T-cell epitopes specific to particular HLA alleles), particularly those that have high concentration of T-cell epitopes [31]. By combining selected sets of short antigen fragments that represent T-cell epitope antigenic diversity, complete sets of viral targets can be covered in a "divide-and-conquer" approach. This may provide a promising basis for multivalent peptide-based vaccines against dengue virus. However, this strategy does not address the dengue virus-specific problem of protection versus immunopathology during secondary infections with a different serotype [2].

Several caveats need to be considered in a study such as this. First, it is well-known that not all HLA-restricted epitopes are 9-mers [32]. This may impact the interpretation of our results, which were based only on 9-mers, and hence may not give a true representation of dengue T-cell epitope antigenic diversity. We selected 9-mers because they represent the typical size of HLA class I T-cell epitopes, as well as the binding core of HLA class II T-cell epitopes [32]. We performed the same analysis with peptides of 8-mers and 10-mers. The results showed no significant difference as compared to the analysis of 9-mers (data not shown).

The second caveat is the sampling bias in dengue virus sequences reported to the public databases. Only dengue sequences that have been studied are reported, and viruses collected in accessible locations, associated with notable disease outbreaks or of known immunological properties are preferentially studied. Consequently, certain dengue proteins have been studied intensively, while the others remained largely unstudied. For example, sequences of the envelope protein, known to be important for immunological activity and viral entry into host [26,33], were the most abundant in our dataset (3183 sequences for all four serotypes), while that of NS4a, which is relatively unknown for immunological activity, was under-represented. In addition, for majority of the proteins, a large portion of the reported sequences were incomplete in length. For example, 95% of DV2 NS5 collected sequences were incomplete in length (data not shown). However, the data used in this study was the most representative available and the large sample size for majority of the proteins helps to decrease the margin of error due to sampling bias. In addition, the reported sequences represent highly pathogenic strains isolated during dengue outbreaks.

There has been no significant increase in the number of unique sequences for dengue virus since the last analysis (December 2005). The September 2006 data set contained a total of 2661 (793 DV1, 784 DV2, 759 DV3 and 325 DV4) dengue unique sequences. This was an increase of 242 unique sequences from the 2005 data set. The increase, approximately 10%, was not expected to significantly affect the results observed for 2005 data set. Therefore, we did not perform the analysis of antigenic diversity on the 2006 data set because of the small increase in the number of unique sequence.

## Conclusion

This study has provided evidence that there are limited numbers of antigenic combinations in variant protein sequences of a viral species and that short regions of the viral proteins are sufficient to capture antigenic diversity of T-cell epitopes. The approach described herein has direct application to the analysis of other viruses, in particular those that show high diversity and/or rapid evolution, such as influenza A virus and human immunodeficiency virus (HIV).

## Methods

### Data collection

All dengue virus protein sequence entries present in the NCBI Entrez protein database [34] were collected in June 2004 and then again in December 2005. Data retrieval was performed through the NCBI taxonomy browser [19] and the respective taxonomy ID for each of the dengue serotypes (DV1-4) are 11053, 11060, 11069 and 11070. The collected entries for both time points were processed separately using identical procedures.

### Data processing: cleaning and grouping

The dengue virus RNA genome is translated into a single polyprotein (~3390 aa) that is cleaved by proteases to yield 10 dengue proteins: the C protein; the M protein, which is synthesized as a larger precursor protein pM; the major E glycoprotein; and seven nonstructural (NS) proteins, NS1, NS2a, NS2b, NS3, NS4a, NS4b and NS5 (Table 2). Individual protein sequences were extracted from collected entries for each DV serotype and grouped according to the 10 dengue proteins for analysis. The protein sequence extraction was done by sequence alignments and identification of known cleavage sites for dengue proteins. The cleavage sites were obtained from the annotation of the GenPept [19] reference polyprotein sequence for each dengue serotype (DV1: AAF59976; DV2: P14340; DV3: AAM51537; DV4: AAG45437) and the literature [35]. The grouping of the extracted sequences for proteins of each serotype was facilitated by local sequence alignment using the BLAST algorithm [36] (parameters: filter – no; expect – 100; descriptions & alignments – 1000), followed by multiple sequence alignment using ClustalX 1.83 [37] with default parameters, followed by manual inspection. Duplicate or identical sequences for proteins within each serotype were removed, and the unique sequences were retained for further analysis. Both full-length and partial unique sequences of each dengue serotype protein were used for the analysis, unless indicated otherwise. Data compiled from public databases are prone to errors and discrepancies [38], which may affect the analysis. Therefore, we inspected the collected DV entries and corrected errors and discrepancies (see additional file 1: Table S1.pdf).

### Protein sequence and antigenic diversity analysis of dengue virus

In the context of this study, protein sequence diversity of a dengue protein was defined as the total number of unique sequences reported in the database for the protein. Sequences having at least a single amino acid difference between them were considered as unique. We calculated the pairwise percentage amino acid identity of the full-length unique sequences of each dengue protein, intra- and inter-serotype, by use of ClustalW 1.83 [39] with default parameters, followed by manual inspection. This was done to survey the extent of amino acid variation and conservation in the latest, comprehensive dengue data of 2005.

Antigenic diversity of a dengue protein was defined in this study as the minimal set of unique sequences required to represent the complete set of overlapping 9-mer peptides encoded by all unique sequences reported in the database for the protein. We developed a bioinformatics method that performs exhaustive search to determine the minimal set for a given protein. The method comprises two steps: (a) generation of a set of overlapping 9-mers from the entire length of all unique sequences reported in the database for the protein, followed by (b) identification of a minimal set of unique sequences that represents all the unique 9-mers. The union of such sets for all the ten proteins of a dengue serotype represents the antigenic diversity of the proteins for the serotype as defined in this study. The computer program for the method was written in Perl and C language.

In the first step of the method, we generated overlapping 9-mers from the entire length of each unique sequence because the whole length was assumed to contain potential targets of T-cell mediated immune responses (T-cell epitopes) [40]. This assumption was based, firstly, on the estimate that from a complete set of overlapping peptides (9 or 10-mers) spanning a protein, on average, 0.1–5% of the peptides will bind to any particular HLA molecule [41]. Secondly, given the large number of HLA molecules (more than 2532 known as of September 2006; [42]), the vast majority of the complete set of overlapping peptides are highly likely to bind at least one molecule from the total HLA pool. Thus, each overlapping peptide is a potential T-cell epitope. This assumption ensures the capture of all possible candidate 9-mer T-cell epitopes that can be present across the entire length of the unique sequence. We focused our antigenic diversity study on 9-mers because they represent the predominant length of HLA class I T-cell epitopes, as well as the binding core of HLA class II T-cell epitopes [32,40]. Furthermore, our preliminary analysis using 8-mers and 10-mers did not produce notably different results compared to the analysis of 9-mers (data not shown). A small number of 9-mers derived from the unique sequences contained unknown residues (denoted by "X") and, hence, were excluded from the analysis because they were antigenically non-informative.

### Determining the effects of sequence determinants on antigenic diversity

The effects on antigenic diversity of two sequence determinants, the number of viral sequences in the studied set and the length of protein antigens were studied. The study was performed on unique sequences from the DV2 envelope protein (retrieved in 2005) because it provided a sufficiently large and well-defined dataset (198 full-length sequences). Test datasets with different numbers of sequences (20, 40, 60, 80, 100, 120 and 140 sequences) and different lengths (23, 46, 128, 138, 276 and 460 aa) were randomly derived from the envelope dataset with repeated sampling (20 repeats). Any duplicate sequences were removed from the test datasets. The minimal set of sequences that represents the complete short-peptide antigenic diversity was determined for each dataset. These minimal sets were used to analyze the effects of the sequence determinants on antigenic diversity.

## List of abbreviations used

DV- Dengue Virus; DV1- Dengue Virus Serotype 1; DV2- Dengue Virus Serotype 2; DV3- Dengue Virus Serotype 3; DV4- Dengue Virus Serotype 4; aa- amino acids; NCBI- National Center for Biotechnology Information; HIV- Human Immunodeficiency Virus; HLA- Human Leukocyte Antigen.

## Competing interests

The author(s) declare that they have no competing interests.

## Authors' contributions

AMK performed the *in silico *experiments and drafter the manuscript. ATH, KXL, KNS, TWT and JTA participated in the design of the study. VB conceived the study, participated in its design and coordination and helped to draft the manuscript. JTA and TWT critically reviewed the manuscript. All authors read and approved the final manuscript.

## Supplementary Material

Additional file 1 Errors and discrepancies found in each dengue serotype (DV1, DV2, DV3 and DV4) data entries collected from the NCBI Entrez protein database. Click here for file

## Figures and Tables

**Table 1:** Collected and unique protein sequences for each dengue serotype in 2004 and 2005 and the corresponding increase in data between the two time points.

Dengue serotype	Data retrieved in 2004 (#)		Data retrieved in 2005 (#)		Increase (#)	
	
	Collected sequences	Unique sequences	Collected sequences	Unique sequences	Collected sequences	Unique sequences
DV1	744	359	2318	724	1574	365
DV2	1426	507	3351	697	1925	190
DV3	597	230	2520	678	1923	448
DV4	932	222	1323	320	391	98

*Total*	*3699*	*1318*	*9512*	*2419*	*5813*	*1101*

**Table 2:** Proteins of a representative dengue virus serotype 2 polyprotein entry (P14340 of 3391 amino acids) in the NCBI Entrez protein database.

Protein	Length (amino acids)
Capsid (C)	114
Precursor membrane (pM)	166
Envelope (E)	495
Nonstructural protein 1 (NS1)	352
Nonstructural protein 2a (NS2a)	218
Nonstructural protein 2b (NS2b)	130
Nonstructural protein 3 (NS3)	618
Nonstructural protein 4a (NS4a)	150
Nonstructural protein 4b (NS4b)	248
Nonstructural protein 5 (NS5)	900

**Table 3:** Unique sequences for the proteins of the four serotypes in 2004 and 2005.

Protein	No. of unique sequences (all four serotypes)	
	
	2004	2005
C	107	196
pM	126	220
E	495	998
NS1	150	224
NS2a	95	142
NS2b	59	78
NS3	80	164
NS4a	37	69
NS4b	57	88
NS5	112	240

*Total*	*1318*	*2419*

**Table 4:** Minimum and maximum percentage sequence identity range for each dengue protein, intra- and inter-serotype.

		DV1	DV2	DV3	DV4	Average PSI			DV1	DV2	DV3	DV4	Average PSI
C	DV1	88–99				65	pM	DV1	92–99				68
	DV2	56–75	81–99					DV2	62–75	79–99			
	DV3	75–84	53–66	91–99				DV3	75–82	60–72	93–99		
	DV4	61–68	57–69	54–60	94–99			DV4	62–67	60–71	64–70	96–99	

E	DV1	89–99				65	NS1	DV1	93–99				72
	DV2	58–70	80–99					DV2	68–75	85–99			
	DV3	72–79	60–69	92–99				DV3	77–80	69–75	94–99		
	DV4	58–66	55–65	61–64	94–99			DV4	67–70	68–73	70–74	93–99	

NS2a	DV1	90–99				39	NS2b	DV1	93–99				60
	DV2	36–40	93–99					DV2	56–62	95–99			
	DV3	43–48	35–40	93–99				DV3	66–70	58–63	96–99		
	DV4	35–39	33–36	36–41	89–99			DV4	56–62	54–59	56–59	94–99	

NS3	DV1	97–99				79	NS4a	DV1	92–99				60
	DV2	78–80	96–99					DV2	56–61	96–99			
	DV3	84–86	79–81	97–99				DV3	63–68	56–63	92–99		
	DV4	75–77	75–77	77–79	97–99			DV4	56–60	59–64	56–62	94–99	

NS4b	DV1	95–99				78	NS5	DV1	96–99				77
	DV2	75–79	95–99					DV2	77–79	95–99			
	DV3	81–85	75–79	97–99				DV3	80–82	77–79	96–99		
	DV4	75–78	77–81	76–79	97–99			DV4	73–76	72–75	74–77	95–99	

The average percentage sequence identities (PSI) are shown for inter-serotype comparisons.

**Table 5:** Reduction of the number of unique dengue sequences by removal of antigenically redundant sequences.

Dengue serotype	Data retrieved in 2004			Data retrieved in 2005		
	
	Unique sequences (#)	Minimal antigenic set		Unique sequences (#)	Minimal antigenic set	
				
		Unique sequences (#)*	Percentage of unique sequences (%)**		Unique sequences (#)*	Percentage of unique sequences (%)**
DV1	359	244	68%	724	493	68%
DV2	507	368	73%	697	466	67%
DV3	230	180	78%	678	482	71%
DV4	222	177	80%	320	243	76%

*Total*	*1318*	*969*	*74%*	*2419*	*1684*	*70%*

*Minimal no. of unique sequences that represent complete short-peptide (9-mer) antigenic diversity of dengue unique sequences reported in NCBI Entrez protein database. **Percentage of unique sequences that represent complete short-peptide (9-mer) antigenic diversity of dengue unique sequences reported in the NCBI Entrez protein database.

**Table 6:** Effects of number of unique dengue virus serotype 2 (DV2) envelope sequences (N) on short-peptide (9-mer) antigenic diversity.

Number of unique sequences (N)	20	40	60	80	100	120	140
Length of sequences	460 aa	460 aa	460 aa	460 aa	460 aa	460 aa	460 aa
Minimal number of unique sequences that represent complete short-peptide antigenic diversity (Mean ± SE)	18 ± 0.30	32 ± 0.54	46 ± 0.70	58 ± 0.87	70 ± 0.87	80 ± 0.87	90 ± 0.71
Percentage of unique sequences that represent complete short-peptide antigenic diversity (%) (Mean ± SE)	90 ± 1.5	80 ± 1.35	77 ± 1.17	73 ± 1.09	70 ± 0.87	67 ± 0.73	64 ± 0.51

The mean and standard error (SE) values are shown for random repeated sampling of 20 times.

**Table 7:** Effect of length of dengue virus serotype 2 (DV2) envelope protein sequences on short-peptide (9-mer) antigenic diversity.

Length of fragments	100% (460 aa)	60% (276 aa)	30% (138 aa)	20% (92 aa)	10% (46 aa)	5% (23 aa)
Number of fragments	187	187	187	187	187	187
Number of unique fragments	187	131	82	58	27	17
Minimal number of fragments that represent complete short-peptide antigenic diversity (Mean ± SE)	111 ± 0.11	74 ± 0.11	48 ± 0.17	38 ± 0.10	24 ± 0.10	14 ± 0.10
Percentage of fragments that represent complete short-peptide antigenic diversity (%) (Mean ± SE)	59 ± 0.06	40 ± 0.06	26 ± 0.09	20 ± 0.05	13 ± 0.05	7 ± 0.05

The mean and standard error (SE) values are shown for random repeated sampling of 20 times.
