# Supplementary material for: Large-scale analysis of antigenic diversity of T-cell epitopes in dengue virus
Source: BMC Bioinformatics. 2006 Dec 18;7(Suppl 5):S4. doi: 10.1186/1471-2105-7-S5-S4 (PMC1764481; doi:10.1186/1471-2105-7-S5-S4)
Supplement: Additional file 1 — Errors and discrepancies found in each dengue serotype (DV1, DV2, DV3 and DV4) data entries collected from the NCBI Entrez protein database. [file 1471-2105-7-S5-S4-S1.pdf]

Table S1: Errors and discrepancies found in each dengue serotype (DV1, DV2, DV3 and DV4) data entries collected from the NCBI Entrez protein database.

DV1 entry	Error/discrepancy description
AAK29447	• The position of the C terminal end of the protein NS4B is mis-annotated. Instead of 2293 it should be 2493. Evidence by sequence similarity to other strains.<br>• The position of the N-terminal of the protein NS5 is mis-annotated. Instead of 2294 it should be 2494. Evidence by sequence similarity to other strains.
A42551	• The C-terminal position of the precursor membrane protein region has been mis-annotated. It should be 205..280 instead of 205..281. Evidence by sequence similarity to other strains.<br>• The N-terminal position of the envelope protein region has been mis-annotated. It should be 281..774 instead of 282..774. Evidence by sequence similarity to other strains.<br>• The C-terminal position of the NS1 protein region has been mis-annotated. It should be 775..1126 instead of 775..1127. Evidence by sequence similarity to other strains.<br>• The N-terminal position of the NS2a protein region has been mis-annotated. It should be 1127..1344 instead of 1128..1344. Evidence by sequence similarity to other strains.
P33478	• The C-terminal position of the NS1 protein region has been mis-annotated. It should be 775..1126 instead of 775..1127. Evidence for this can be found in Fu *et al*., (1992).<br>• The N-terminal position of the NS2a protein region has been mis-annotated. It should be 1127..1344 instead of 1128..1344. Evidence for this can be found in Fu *et al*., (1992).
AAN03445	• Under the field “Features”, we see the following statement:<br>Protein 1..3392<br>/product="envelope glycoprotein"<br>The statement is not correct because envelope glycoprotein is only a part of the whole polyprotein. Instead of envelope glycoprotein it should have been “polyprotein” as in entry AAO47361 (GI:34596500)
AAB70694	• The N-terminal position of the capsid protein region has been mis-annotated. It should be 1..114 instead of 2..114. Evidence by sequence similarity to other strains.
AAB70696	• The N-terminal position of the capsid protein region has been mis-annotated. It should be 1..114 instead of 2..114. Evidence by sequence similarity to other strains.<br>• The C-terminal position of the precursor membrane protein region has been mis-annotated. It should be 115..280 instead of 115..278. Evidence by sequence similarity to other strains.<br>• The C-terminal position of the mature membrane protein region has been mis-annotated. It should be 206..280 instead of 206..278. Evidence by sequence similarity to other strains.<br>• The N-terminal position of the envelope protein region has been mis-annotated. It should be 281..775 instead of 279..775.

	Evidence by sequence similarity to other strains.
AAB70695	• The N-terminal position of the capsid protein region has been mis-annotated. It should be 1..114 instead of 2..114. Evidence by sequence similarity to other strains.<br>• The C-terminal position of the precursor membrane protein region has been mis-annotated. It should be 115..280 instead of 115..278. Evidence by sequence similarity to other strains.<br>• The C-terminal position of the mature membrane protein region has been mis-annotated. It should be 206..280 instead of 206..278. Evidence by sequence similarity to other strains.<br>• The N-terminal position of envelope protein region has been mis-annotated. It should be 281..775 instead of 279..775. Evidence by sequence similarity to other strains.
**DV2 entry**	**Error/discrepancy description**
AAL00888	• The position of the C-terminal of the capsid protein is mis-annotated. It should be 1..114 instead of 1..150. Evidence by sequence similarity to other strains.<br>• The position of the N-terminal of the precursor membrane protein is mis-annotated. It should be 115..280 instead of 151..280. Evidence by sequence similarity to other strains.
CAD31751	• The protein 115..280 should be annotated as prM protein instead of envelope protein.<br>• The protein 281..774 should be annotated as envelope protein instead of prM protein.<br>• The position of the C-terminal of the envelope protein is mis-annotated. It should be 281..775 instead of 281..774. Evidence by sequence similarity to other strains.<br>• The position of the N-terminal of the NS1 protein is mis-annotated. It should be 776..1127 instead of 775..1127. Evidence by sequence similarity to other strains.<br>• The position of the C-terminal of the envelope protein is mis-annotated. It should be 1346..1475 instead of 1346..1474. Evidence by sequence similarity to other strains.<br>• The position of the N-terminal of the NS3 protein is mis-annotated. It should be 1476..2093 instead of 1475..2093. Evidence by sequence similarity to other strains.
AAA42941	• The N-terminal position of the capsid protein region has been mis-annotated. It should be 1..114 instead of 2..114. Evidence by sequence similarity to other strains.<br>• The position of the C-terminal of the NS4a protein is mis-annotated. It should be 2094..2243 instead of 2094..2379. Evidence by sequence similarity to other strains.<br>• The position of the N-terminal of the NS4b protein is mis-annotated. It should be 2244..2491 instead of 2380..2491. Evidence by sequence similarity to other strains.
**DV3 entry**	**Error/discrepancy description**
P27915, AAA99437 and GNWVD3	• All the three entries have NS1/NS2a and NS4a/NS4b junctions mis-annotated; the amino acid sequence at these junctions in the three entries were not similar to those

	described in Osatomi *et al.* (1990).
**DV4 entry**	**Error/discrepancy description**
AAB28474	• The position of the C-terminal of the capsid protein is mis-annotated. It should be 1..113 instead of 1..110. Evidence by sequence similarity to other strains.<br>• The position of the N-terminal of the precursor membrane protein is mis-annotated. It should be 114..279 instead of 112..279. Evidence by sequence similarity to other strains.
AAA42964	• The position of the C-terminal of the envelope protein is mis-annotated. It should be 280..774 instead of 280..733. Evidence by sequence similarity to other strains.<br>• The position of both the N and C termini of the NS1 protein is mis-annotated. It should be 775..1126 instead of 734..1184. Evidence by sequence similarity to other strains.<br>• The position of both the N and C termini of the NS2a protein is mis-annotated. It should be 1127..1344 instead of 1185..1343. Evidence by sequence similarity to other strains.<br>• The position of both the N and C termini of the NS2b protein is mis-annotated. It should be 1345..1474 instead of 1344..1473. Evidence by sequence similarity to other strains.<br>• The position of both the N and C termini of the NS3 protein is mis-annotated. It should be 1475..2092 instead of 1474..2091. Evidence by sequence similarity to other strains.<br>• The position of both the N and C termini of the NS4a protein is mis-annotated. It should be 2093..2242 instead of 2092..2374. Evidence by sequence similarity to other strains.<br>• The position of both the N and C termini of the NS4b protein is mis-annotated. It should be 2243..2487 instead of 2092..2374. Evidence by sequence similarity to other strains.<br>• The position of both the N and C termini of the NS5 protein is mis-annotated. It should be 2488..3387 instead of 2487..3386. Evidence by sequence similarity to other strains.
P09866	• The position of the C-terminus of the NS1 protein is mis-annotated. It should be 775..1126 instead of 775..1185. Evidence by sequence similarity to other strains.<br>• The position of the N-terminus of the NS2a protein is mis-annotated. It should be 1127..1344 instead of 1186..1344. Evidence by sequence similarity to other strains.<br>• The position of the C-terminus of the NS4a protein is mis-annotated. It should be 2093..2242 instead of 2093..2375. Evidence by sequence similarity to other strains.<br>• The position of the N-terminus of the NS4b protein is mis-annotated. It should be 2243..2487 instead of 2376..2487. Evidence by sequence similarity to other strains.
GNWVDF	• The position of the C-terminus of the NS1 protein is mis-annotated. It should be 774..1125 instead of 774..1184. Evidence by sequence similarity to other strains.<br>• The position of the N-terminus of the NS2a protein is mis-annotated. It should be 1126..1343 instead of 1185..1343.

	Evidence by sequence similarity to other strains.<br>• The position of the C-terminus of the NS4a protein is mis-annotated. It should be 2092..2241 instead of 2092..2374. Evidence by sequence similarity to other strains.<br>• The position of the N-terminus of the NS4b protein is mis-annotated. It should be 2242..2486 instead of 2375..2486. Evidence by sequence similarity to other strains.

References

Osatomi K, Sumiyoshi H: **Complete nucleotide sequence of dengue type 3 virus genome RNA**. *Virology* 1990, **176**(2):643-647.

Fu J, Tan BH, Yap EH, Chan YC, Tan YH: **Full-length cDNA sequence of dengue type 1 virus (Singapore strain S275/90)**. *Virology* 1992, **188**(2):953-958.
